# Photoperiod regulate gonad development via kisspeptin/kissr in hypothalamus and saccus vasculosus of Atlantic salmon (*Salmo salar*)

**DOI:** 10.1371/journal.pone.0169569

**Published:** 2017-02-15

**Authors:** Liang Chi, Xian Li, Qinghua Liu, Ying Liu

**Affiliations:** 1 Center of Biotechnology R&D, Institute of Oceanology, Chinese Academy of Sciences, Qingdao, P.R. China; 2 College of Animal Science and Technology, Qingdao Agricultural University, Qingdao, P.R. China; 3 National & Local Joint Engineering Laboratory of Ecological Mari culture, Qingdao, China; John Hopkins University School of Medicine, UNITED STATES

## Abstract

Atlantic salmon exhibit seasonal reproduction. However, the mechanisms governing this are still unclear. Generally speaking, kisspeptin has been recognized as a regulator of reproduction. Here, we report a relationship between kisspeptin, GnRH and photoperiod in Atlantic salmon. The results demonstrated that the expression of the *Atlantic salmon* kisspeptin-receptor (s*kissr*) was not always consistent with the expression pattern of Atlantic salmon *GnRH3* (*sGnRH3*) during all developmental processes. Kisspeptin may exert its influence primarily in the early and later stages of gonad development by promoting the secretion of *sGnRH3*. Meanwhile, the expression levels of *kissr* were higher in fish with gonads at stage II and stage V under the long-day photoperiod regime than under the short-day regime. In addition, both s*kissr* and *sGnRH3* were also expressed in the saccus vasculosus (SV), an organ only found in fish. The SV might be a seasonal sensor regulating reproduction in addition to the hypothalamus (Hyp).

## Introduction

Seasonal reproduction is an important adaptive trait for animals living outside the tropics and photoperiod (day length) is undoubtedly one of the most effective environmental signals available to living organisms, including fish [1, 2]. Photoperiod is the only environmental factor which provides a reliable indicator of the time of year and so enables reproduction and growth processes to be expressed at the most appropriate date [3]. It is now widely accepted that seasonally changing photoperiods provide the proximate environmental signal for the initiation and co-ordination of gonad development in most temperate fish [4].

In mammals, the synchronization of reproduction with photoperiod is mediated by melatonin which is secreted by the pineal organ[5, 6]. Melatonin is thought to stimulate the production of kisspeptin and type 2 iodothyronine deiodinase in the pars tuberalis (PT) of the pituitary gland [7–11]. Teleost fish, do not possess a distinct PT, and the signal transduction pathway for their reproduction remains unclear [12].

Kisspeptin and its receptor GPR54 (kissr) have been identified as key factors in controlling the reproductive cycle by stimulating gonadotropin releasing hormone (GnRH) secretion in mammals. Loss of functional mutations in mouse and human GPR54 show that GPR54 is absolutely required for reproduction in mammals [13–15]. In teleost, the study of kisspeptin and its receptor is still in its infancy. Parhar and co-workers were the first to report the importance of kissr and GnRH in fish reproduction and found that kissr was involved in the sexual development of a cichlid fish[16]. Since then, kisspeptin and kissr have become the subject of active research in fish reproduction. It is believed that kisspeptin and kissr perform similar roles in fish to kisspeptin/GPR54 reported in mammals. Kisspeptin has been reportedly associated with the onset of puberty in some fish species [17–19], and has GnRH regulatory functions in several others [20]. In addition, Martinez-Chavez et al. found that a long photoperiod could delay the onset of puberty and suppress *GPR54* expression in Nile tilapia by reducing the expression of kisspeptin [17]. This was the first study of teleost to suggest a possible connection between photoperiod and kisspeptin. Similar results have also been found in other fish species [21–23]. Furthermore, most research into photoperiods and GnRH have focused on the stage of puberty. Furthermore, there is very limited reference to the effects of kisspeptin/kissr on other stages of gonad development [24, 25].

In mammals and birds, the PT of the pituitary gland is considered to play a key role in the regulation of seasonal reproduction [26–28]. However, fish differ from mammals and birds in not processing an anatomically distinct PT [12], and researchers have considered the hypothalamus to be the regulatory hub of photoperiodism in fish. Meanwhile, kisspeptin genes are expressed in the nucleus ventralis tuberis (NVT) and the nucleus posteriors periventricularis (NPPv) of the hypothalamus in the medaka [29, 30]. This suggests that kisspeptin produced in the hypothalamus mediates seasonal reproduction[22].

A recent study of masu salmon (*Oncorhynchus masou masou*) found that some important factors involved in seasonal reproduction, such as thyroid-stimulating hormone (TSH), TSH-receptor and thyroid hormone-activating enzymes (DIO), are expressed in the saccus vasculosus (SV) [12]. The SV is an organ unique to fish, which is located posterior to the pituitary gland on the floor of the hypothalamus. This finding provides a new means of understanding the functions of the SV and the regulation of seasonal reproduction.

Atlantic salmon (*Salmo salar* L), are native to the North Atlantic and its surrounding rivers, and were introduced into Chinese using Recirculating Aquaculture Systems (RAS). Atlantic salmon are short-day seasonal breeders and are very sensitive to the photoperiod[31]. Furthermore, salmonids are characterized by their direct sensitivity to daylight and lack of endogenous rhythms found in other fish [32, 33]. In a previous study, we found that photoperiod can significantly affect the gonadal development of Atlantic salmon reared in RAS. In order to enrich our knowledge of the functions of the SV in teleost fish, we set out to clarify the relationship between *kissr* and *GnRH* in the Hypothalamus and SV of Atlantic salmon under the different photoperiod regimes.

## Materials and methods

### Experimental design

Atlantic salmon with an average body mass of 1071.70 ± 155.54g were purchased from the Shandong Oriental Ocean Sci-Tech Co., Ltd, Shandong province, China. The fish were then allowed to acclimate for four weeks in a RAS under 24L:0D photoperiod, after which they were distributed between the experimental RAS tanks (130 cm height ×200 cm diameter). Each experimental group contained 60 fish. During the experimental period, the water temperature was maintained at 16.27 ± 0.54°C, pH 7.2–7.5, total ammonia-nitrogen <0.25mg/L, and salinity 24–26 over the course of the experiment.

The fish were divided into six photoperiod treatment groups. Four of the photoperiod treatments remained constant throughout the experiment 24L:0D, 18L:6D, 12L:12D, 16L:8D. The remaining two photoperiod groups had varying photoperiods during the experiment. In the first treatment, the photoperiod changed from 24L:0D to 8L:16D (the LL-SL treatment), and in the second the photoperiod changed from 8L:16D to 24L:0D (the SL-LL treatment), the photoperiod being changed by five minutes per day in both cases. There were three replicate tanks (initially n = 60 fish/tank). The experiment was performed over a seven month period from September to the following March, a period spanning the first reproductive period. Fish were sampled every month. Nine female fish were anesthetized until death in seawater with 0.05% MS-222 (3-Aminobenzoic acid ethyl ester methanesulfonate). Body weight, length and gonad weight were recorded, and the brains were immediately, weighed, frozen in liquid nitrogen, and stored at -80°C. The gonads and brains were placed in Bouin’s solution for 24h and then stored in 70% ethanol for later histological examination. Blood was centrifuged and plasma was stored at -80°C.

Furthermore, the gonadal development in this paper were determined based on GSI, external morphological and histology observation as follow: stage II (beginning of vitellogenesis with primary yolk vesicles,GSI = 0.15%±0.05%),stage III (secondary yolk stage, GSI = 0.31% ± 0.18%), stage IV (accumulating of yolk happened, GSI = 11.87% ± 8.85%), stage V(cytoplasm of oocyte was filled with yolk granules, GSI = 17.57%±3.01%). All of the procedures described in this study were reviewed and approved by the ethical committee of the Institute of Oceanology, Chinese Academy of Sciences.

### RNA extraction, preparation of first strand cDNA and Quantitative real-time PCR

Total RNA was extracted from the different regions of the Atlantic salmon brains (telencephalon, diencephalon, Hyp, mesencephalon and the SV) using a fast 200 RNA extraction kit (Fastagen, Shanghai, China), according to the manufacturer’s instructions. The total RNA was then dissolved into 20 μL RNase free water. After that, 1μg of total RNA was reverse transcribed to first-strand cDNA by a First- Strand cDNA Synthesis SuperMix (TransGen, Beijing, China) according to the manufacturer’s instructions. The reaction system contained 2 × TS Reaction Mix, 0.5μL Oligo dT Primer, 1μL genomic DNA remover and RNase free water up to a 20μL volume.

The primers used to amplify *sGnRH3*, *skissr* and *β-actin* ([34] were described in Table 1, which were designed from conserved regions of fish *GnRH3* and *kiss2r* in the GenBank database.

**Table 1:** The primers used for amplification of gene by PCR.

Primers	sequences	Annealing temperature (°C)
***GnRH3***	F: 5’ GTGGTGGTGTTGGCGTTGGTAG 3’	59
	R: 5’ TAGTGATGCTGAATGTCTGCTTG3’	
***kissr***	F: 5’ GGAHCTYCANCANCYCMAMCDCAC 3’	58
	R:5’CATGGYYTAKWTCTCTCWKGVCDTWG3’	
***β-actin***	F: 5’ GACGCGACCTCACAGACTACCT3’	58
	R: 5’ CGTGGATACCGCAAGACTCCATAC3’	

Note: F: forward primer; R: reverse primer

The gene expression of *sGnRH3* and *skissr* were quantified using SYBR TransStart Top Green qPCR SuperMix Kit (TransGen, Beijing, China) in an eppendorf Mastercycler ep realplex real-time PCR instrument (Eppendorf, German), using the standard curve method with *β-actin* as a reference gene. The primers used to amplify *sGnRH*, *skissr*, and *β-actin* are listed in Table 2. Amplification was performed in a 20 μL reaction volume according to the manufacturer’s instructions, using 10 μL 2×Top Green qPCR SuperMix, 0.4μL (4μM) forward and reverse primers, 0.4μL Passive Reference Dye, 1μL cDNA and ddH_2_O up to a 20 μL final volume.

**Table 2:** The primers used for real-time RT-PCR.

Genes	Sequences of primers	Products (bp)
***GnRH3***	F: 5’- CACTGGTCGTATGGCTGGCTAC-3’	245
	R: 5- TAGTGATGCTGAATGTCTGCTTG-3	
***kissr***	F:5’-GAGGGCTACTGGTATGGACCGAGACA-3’	284
	R:5’-CCCCAGCAGATGGTGAATAAGAGGAC-3’	
***β-actin***	F: 5’- ATCCACGAGACCACCTACAACTCC -3’	268
	R: 5’- CGTACTCCTGCTTGCTGATCCAC-3’	

Note: F: forward primer; R: reverse primer

### Double color fluorescence *in situ hybridization* for Atlantic salmon brains

Antisense digoxigenin (DIG) probes and antisense fluorescein isothiocyanate (FITC) were transcribed for salmon *sGnRH* gene and *skissr* gene by using a DIG and FITC-labeling Kit (Roche, US), Sense probe for salmon s*GnRH* and *skissr* were transcribed as negative control (Table 3).

**Table 3:** Primers use for *in situ* hybridization.

Genes	Sequences
***GnRH3***	Antisense:5’ CAGGTGGTGGTGTTGGCGTTGGTAG 3’
	Sense:5’ AAATGTGATGTTTGTTGGAAATGGA 3’
***kissr***	Antisense:5’ AGGGCTACTGGTATGGACCGAGACA 3’
	Sense:5’ ACTGGAACAGGGCGAAGAGTTGGAT 3

Note: F: forward primer; R: reverse primer

The brains from Atlantic salmon reared in the different photoperiod treatments were fixed in 4% paraformaldehyde in 0.1 M PBS (phosphate buffered saline, pH 7.4) at 4°Covernight. The samples were then dehydrated using a graded methanol series. After that, the samples were mixed with warm paraffin to embed. Sections of paraffin embedded brains were prepared on 5μM glass slides coated with 0.1% Poly-L-lysine solution. The partial CDS of *sGnRH3* and *skissr* were cloned into pGEM-T vectors for preparing sense and antisense RNA probes from a T7 or SP6 promoter using a FITC or digoxigenin (DIG) RNA Labeling Kit (Roche). The sections were hybridized with the sense or antisense probes at 66°C for 18 hours. After hybridization, the samples were incubated overnight at 4°C with horseradish peroxidase (POD)-conjugated anti-FITC-antibody (Roche) at a 1:2000 dilution in the blocking solution to detect the FITC signal. After three washes in PBST, the samples were incubated for one hour in TSA-Fluorescein at a 1:150 dilution in TSA Amplification Buffer. The samples were then subjected to detect the DIG signal. They were incubated overnight at 4°C with POD-conjugated anti-DIG antibody (Roche) at a 1:2000 dilution in blocking buffer with 1% H_2_O_2_. Following three PBST washes, the samples were incubated in TSA-Plus Tetramethylrhodamine for one hour. Double color fluorescence *in situ* hybridization was performed using tyramide signal amplification TSA^™^ Plus Fluorescein & Tetramethylrhodamine (TMR), according to the manufacturer’s instructions (NEL756, PerkinElmer). The nuclei were stained using 4’-6-Diamidino-2-phenylindole (DAPI) and embedded in ProLong^®^ Gold Anti-fade reagent (Invitrogen). The slides were then mounted and photographed using a Nikon Eclipse 50i fluorescence microscope. In this procedure, two antisense RNA probes were co-incubated in a single sample during the hybridization step, and developed red and green fluorescence.

### Histology

The fixed specimens were dehydrated in graded series of alcohol and embedded in paraffin. Paraffin samples were cut in series of sagittal and cross-sections (5 μm), then stained hematoxylin and eosin (H&E) for histological observation under a light microscope (NikonYS-100, Tokyo, Japan) and the pictures were taken with a digital camera (Nikon coolpix-4500, Tokyo, Japan).

### Statistical analyses

All statistical analyses were performed using SPSS 20.0. The results were presented as means ± SD and compared using a one-way ANOVA followed by Tukey’s test. All assays were performed in independent triplicates.

## Results

### The location of *skissr* and *sGnRH3* in the brain of Atlantic salmon

**Fig 1:**
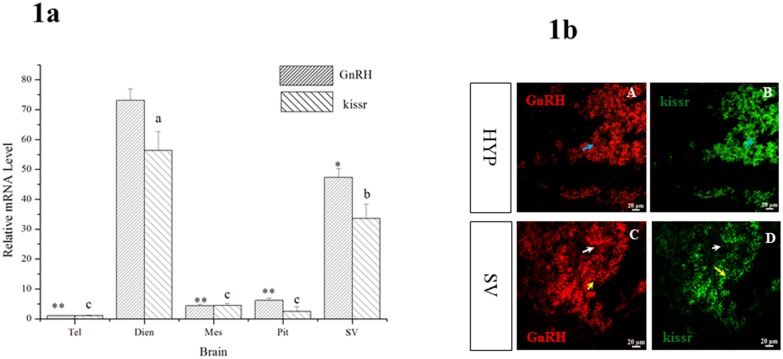
1a: The distribution of *sGnRH3* and *skissr* in the Atlantic salmon brain. Tel: telencephalon; Dien: diencephalon; Mes: mesencephalon; Pit: pituitary gland; SV: saccus vasculosus. Fig 1b: The expression of *sGnRH3* and *skissr* in the hypothalamus (A, B) and SV (C, D). The cells which express *sGnRH3* mRNA are indicated by blue arrows; the white arrows indicate the supporting cells of the SV; and the yellow arrows indicate the coronet cells of the SV. The results show that *skissr* is mainly expressed in the cerebrospinal fluid-contacting (CSF-c) cells of the SV. Hyp: hypothalamus; SV: saccus vasculosus; Results are presented as mean ± SD. Significant differences were found at p≦0.5* and p≦0.1**; and different letters indicate statistical significance at p < 0.05.

First, the mRNA expressions of *skissr* and *sGnRH3* were detected in the different regions of the Atlantic salmon brain using qPCR (Quantitative real-time PCR) with *β-actin* mRNA as a reference gene. The results showed that the both *sGnRH3* and s*kissr* transcripts were primarily expressed in the diencephalon. The transcription levels of *sGnRH3* and s*kissr* were higher in the SV than in other parts of the brain except the diencephalon (Fig 1a.).

Second, the diencephalon and SV of Atlantic salmon were isolated and used to perform *in situ hybridization* in order to confirm the precise location of *sGnRH3* and *skissr* transcripts. The results showed that *sGnRH3* and *skissr* transcripts were mainly expressed in the Hyp of the diencephalon. In the SV, the *sGnRH3* and *skissr* also showed the same expression pattern. Both *sGnRH3* and *skissr* were found in the cells close to the ventricles (Fig 1b).

### Changes in *skissr* in the Hyp and SV during gonad development

**Fig 2:**
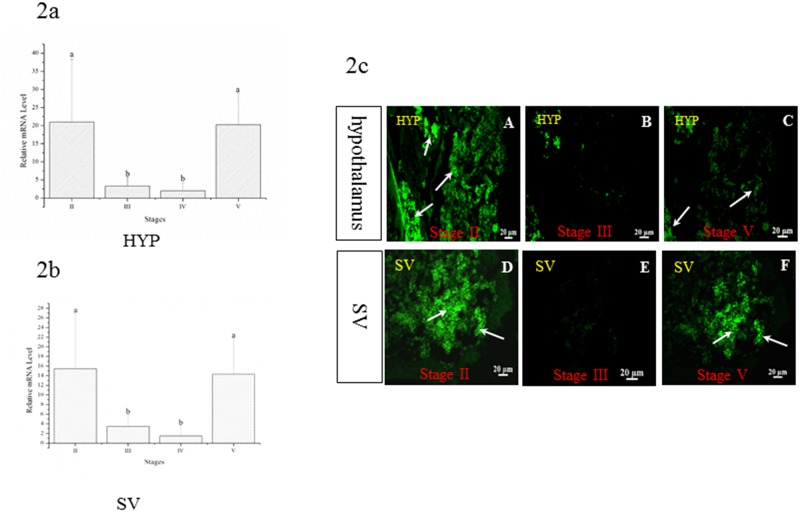
The expression pattern of *skissr* in the hypothalamus (2a) and SV (2b) during the development process of Atlantic salmon. II: the gonad at stage II; III: the gonad at stage III; IV: the gonad at stage IV; V: the gonad at stage V. Fig 2c: The expression of *kissr* in the Atlantic salmon hypothalamus (A, B, C) and SV (D, E, F) when fish with different stages of gonad development are assayed using *in situ* hybridization. SV: Saccus vasculosus. The *kissr* was mainly expressed in the early and late stages of gonad development in Atlantic salmon both in the hypothalamus and the SV. Data are shown as mean ±SD. One-way ANOVA is performed to determine the significant differences between means Columns sharing different letters show significant difference (p < 0.05).

The experiment ran throughout virtually every stage of development of the Atlantic salmon, from stage II to maturity. The expression levels of *kissr* were taken as the mean value in each photoperiod treatment. In the early and late stages of gonad development, when the ovaries were at stage II and stage V, the expression levels of *kissr* transcripts were significantly higher than at stage III and stage IV (Fig 2a and 2c). A similar phenomenon was also observed in the SV (Fig 2b and 2c).

### Changes in *sGnRH3* in the Hyp and SV during gonadal development

**Fig 3:**
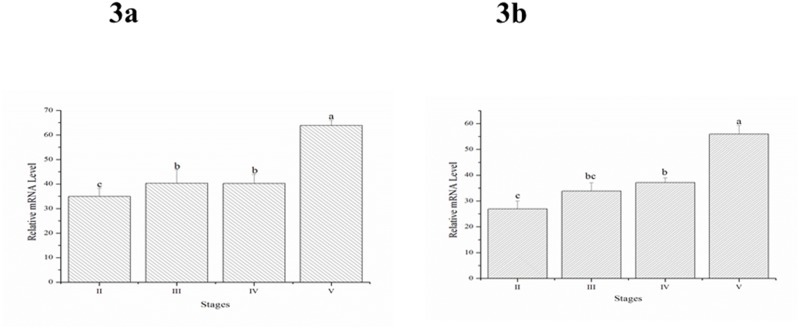
The expression pattern of *sGnRH3* in the hypothalamus (3a) and saccus vasculosus(3b) during the development of Atlantic salmon. II: the gonad at stage II; III: the gonad at stage III; IV: the gonad at stage IV; V: the gonad at stage V.

The expression of *sGnRH3* in the Hyp and SV increased as the gonads developed. The expression of *sGnRH3* transcripts was lowest when the ovaries were at stage II. The expression of *sGnRH3* mRNA was relatively stable when the ovaries were at stage III and stage IV. The mRNA level of *sGnRH3* increased to its highest when the ovaries were at stage V (Fig 3a). The level of *sGnRH3* transcripts in the SV showed the same expression pattern as in the Hyp, while the expression level of *sGnRH3* in the SV was lower than in the Hyp (Fig 3b)

### The expression pattern of *skissr* under different photoperiod treatments in the Hyp and SV of Atlantic salmon

**Fig 4:**
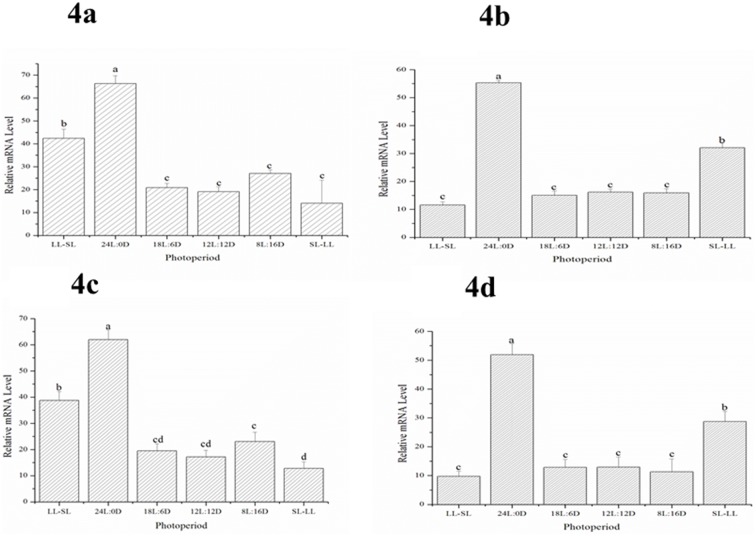
The expression levels of *skissr* transcripts in the hypothalamus (Fig 4a and 4c) and saccus vasculosus (Fig 4b and 4d) in fish with gonads at stage II (Fig 4a and 4b) and stage V (Fig 4c and 4d) under different photoperiod treatments. Data are shown as mean ±SD. One-way ANOVA is performed to determine the significant differences between means Columns sharing different letters show significant difference (p < 0.05).

Due to the s*kissr* was mainly expressed during the early and later stages of gonad development in Atlantic salmon. So, we examined the expression of s*kissr* in the Hyp and SV under the different photoperiod treatments, when fish ovaries at stage II and stage V. The results showed that photoperiod can affect the expression of s*kissr* both in the Hyp and SV (Fig 4). When the ovaries were at stage II, s*kissr* transcript levels were highest in the 24L:0D photoperiod treatment, followed by the LL-SL treatment (Fig 4a) these being the two treatments with the longest photoperiods. There were no significant differences between the other photoperiod groups. When the ovaries were at stage V, the highest *kissr* transcript levels were detected in the 24L:0D group followed by the LL-SL group (Fig 4c). There were no significant differences between other photoperiod treatments. The expression pattern of *skissr* in the SV was similar to that found in the Hyp, and the level of *skissr* transcripts in the SV was lower than in the Hyp (Fig 4b and 4d).

### The expression pattern of *sGnRH3* under different photoperiods in the Hyp and SV of Atlantic salmon

**Fig 5:**
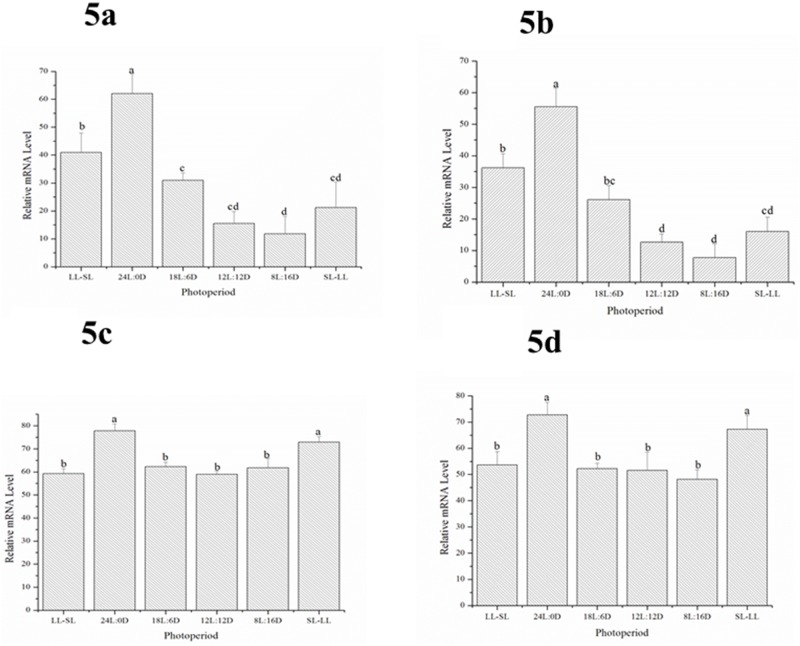
The expression levels of *sGnRH3* transcripts in the hypothalamus (Fig 5a and 5c) and saccus vasculosus (Fig 5b and 5d) in fish with gonads at stage II (Fig 5a and 5b) and stage V (Fig 5c and 5d) under the different photoperiod treatments. Data are shown as mean ±SD. One-way ANOVA is performed to determine the significant differences between means Columns sharing different letters show significant difference (p < 0.05).

The expression levels of *sGnRH3* were also affected by the photoperiod. When the ovaries were at stage II, the highest expression levels of *sGnRH3* were detected in the 24L:0D photoperiod treatment, followed by the LL-SL treatment. The lowest expression levels were observed in the 8L:16D treatment, followed by 12L:12D and SL-LL (Fig 5a). However, when the ovaries were at stage IV, there were no significant differences between the treatments. In addition, when the ovaries were at stage V, the expression levels of *sGnRH3* transcripts were once more affected by the photoperiod. The highest expression levels of *sGnRH3* transcripts were detected in the 24L:0D and SL-LL treatments, and there were no significant differences between the others (Fig 5c). The expression patterns of *sGnRH3* in the SV were similar to those in the Hyp (Fig 5b and 5d).

### The co-expression of *sGnRH3* and *skissr* in the Hyp and SV of Atlantic salmon

**Fig 6:**
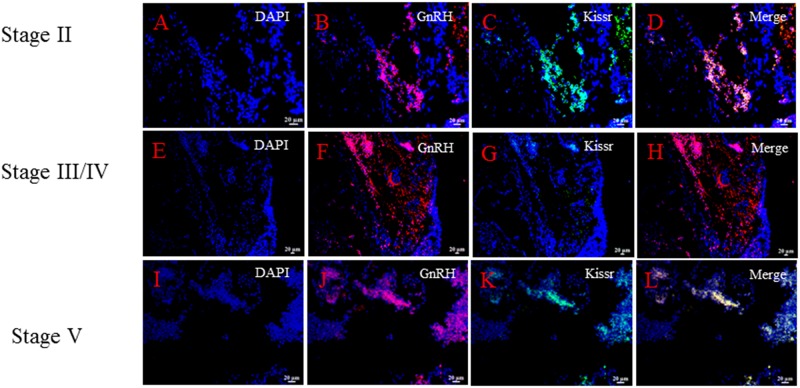
The co-expression of *sGnRH3* and *kissr* in the hypothalamus during the different stages of gonad development. A-D: the co-expression pattern of sGnRH3 and *skissr* in fish with gonad at stage II; E-H: the co-expression pattern of *sGnRH3* and *skissr* in fish with gonads at stage III/ IV (at this time, the transcript levels of *kissr* are very low); I-L: the co-expression pattern of *sGnRH3* and *skissr* in fish with gonads at stage V.

**Fig 7:**
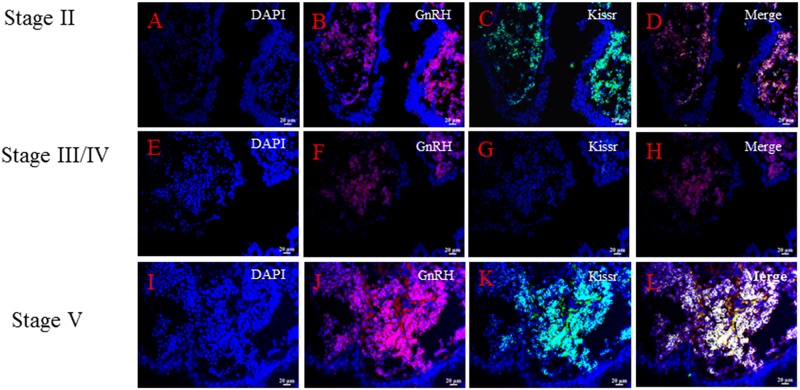
The co-expression of *sGnRH3* and *skissr* in the saccus vasculosus during the different gonad stages. The co-expression in the SV is similar to that in the hypothalamus. A-D: the co-expression pattern of *sGnRH3* and *skissr* in fish with gonads at stage II; E-H: The co-expression pattern of *sGnRH3* and *skissr* in fish with gonads at stage III/ IV; I-L: The co-expression pattern of *sGnRH3* and *skissr* in fish with gonads at stage V.

In order to investigate the possible relationship between GnRH neuron and kisspeptin in Atlantic salmon, the co-expression of *sGnRH3* and *skissr* transcripts were examined in both the Hyp and SV. The results showed that the cells co-express both sGnRH3 and s*kissr* transcripts both in the Hyp and SV. Meanwhile, this co-expression pattern generally appeared when the ovaries were at stages II and V, there was no detectable co-expression in the other two stages. (Figs 6 and 7).

## Discussion

Reproduction process is achieved through a precise synchronization of gonadal development and environmental signals [4]. So far, many environmental factors have been found to affect reproduction, including photoperiod, temperature, nutritional status, rainfall, population level and lunar phase [35]. Of these factors, it is widely accepted that photoperiod and temperature are the only environmental factors that provide a consistent guide to the timing of reproduction, and that photoperiod provides the most precise and reliable signal to entrain the reproduction process [36]. In our previous study on Atlantic salmon (in press), we also found that photoperiod could affect gonadal development, and that a long photoperiod could promote gonadal development.

Kisspeptin has been identified as playing a key role in the initiation of puberty and the regulation of seasonal breeding in mammals [11, 37, 38]. Research into kisspeptin in teleost fish is still in its infancy, and it is speculated that kisspeptin performs similar roles in fish species as in mammals.

In this study, we first investigated the relationship between *sGnRH3* and *skissr*. *sKissr* was mainly expressed in fish during the early and late stages of gonad development.. However, the *sGnRH3* transcript levels did increase as gonad development progressed. Research on the effect of kisspeptin on gonad development after puberty is very limited. Only a few studies shown that kisspeptin can affect ovulation during the reproductive stages [21, 39]. However, results regarding the role of kisspeptin in gonad development are still contradictory. Some researchers believe that kisspeptin can promote the secretion of luteinizing hormone (LH) [40]. On the other hand, another study found that a similar dose of kisspeptin cannot elicit LH secretion[41]. In light of the results of this study, we speculated that kisspeptin can promote *GnRH* release only in the early and late stages of gonadal development. In order to confirm this hypothesis, the co-expression of *sGnRH3* and *skissr* mRNA in the brain at the different gonad stages is examined using double color fluorescence *in situ* hybridization. The results showed that the *skissr* is expressed in the *sGnRH3* neurons, indicating that kisspeptin might affect *GnRH* secretion directly. The *skissr* transcripts mainly appeared at stage II and stage V. We therefore speculated that kisspeptin initiates *GnRH* release at two stages, the early stage including puberty, and the later stage of gonadal development. Both of these periods are critical to reproduction, and we believe that these two stages need more *GnRH* to ensure that the gonads develop. Zmora *et al*. reported that kisspeptin regulated *GnRH* release is stage dependent especially at the pre-spawning phase in striped bass (*Morone saxatilis*) [19, 39]. In seasonally breeding mammals, some researchers implied that the photoperiodic control of reproduction may involve indirect/direct regulation of the kisspeptin/kissr system [10, 42, 43]. In teleost, there are very few studies of photoperiod and the kisspeptin/kissr system. Martinez-Chavez reported that a long photoperiod could inhibit the kisspeptin receptor expression and then delay the onset of puberty[17]. However, another study in medaka found that a long photoperiod induced higher numbers of kiss neurons than short photoperiods [29]. There is still no direct evidence as to whether photoperiod can regulate kisspeptin expression. In this study, we found that *kissr* expression was controlled by the photoperiod when fish had gonads at stages II and V. When fish had gonads at stage II, the higher levels of *skissr* transcripts were mainly apparent in the LL-SL and 24L:0D treatments. At this time, the photoperiod of the LL-SL group was about 21L:3D, also a long-day photoperiod. Fish with gonads at stage V mainly showed the higher level of *kissr* transcripts in the 24L:0D and in the SL-LL photoperiod treatments, while the SL-LL photoperiod was changing from short-day to long-day. These results indicate that a long photoperiod can promote the expression of *kisspeptin* and *kissr*. In addition, we detected changes in *GnRH* transcripts under different photoperiods when the fish gonads were at stage II and stage V. The *sGnRH3* transcript levels were also higher under the long-day photoperiod, similar to the changes in *skissr* transcript levels. Combined with the results from double-color *in situ* hybridization, we conclude that long-day photoperiods can promote *sGnRH3* secretion via the kisspeptin/kissr system only at both early and later stages of gonad development.

**Fig 8:**
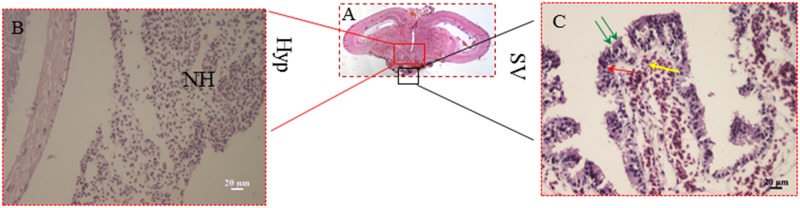
The histology of the Atlantic salmon hypothalamus and saccus vasculosus in Atlantic salmon; A: longitudinal section of the Atlantic salmon brain. B: enlarged picture of the hypothalamus; C: enlarged picture of the saccus vasculosus. Hyp: hypothalamus; SV: saccus vasculosus. Green arrows indicate the coronet cells; the red arrow indicates the supporting cells; and the yellow arrow indicates the cerebrospinal fluid- contacting cells. NH: neurohypophysis.

The SV is a circumventricular organ of the Hyp of fish, and the functions of the SV have not yet been entirely clarified[44]. A recent study of masu salmon (*O*. *masou masou*) found that some elements controlling seasonal reproduction are expressed in the SV, and that ablation of the SV prevents photoperiodically-induced gonadal development. This suggests that the SV plays a key role as a seasonal sensor in fish [12, 45]. In order to investigate whether the SV is an organ that can regulate seasonal reproduction via the kisspeptin/kissr system in Atlantic salmon, the changes in expression of *skissr* and *sGnRH3* under different photoperiod treatments were examined. The SV is composed of coronet cells, supporting cells and cerebrospinal fluid contacting cells (Fig 8) [46]. We found that both *skissr* and *sGnRH3* were expressed in cells close to the ventricles, which might be the CSF-c cells (not in the coronet cells) of the SV. This indicates that SV might function to regulate gonad development. The changes in *sGnRH3* and *skissr* transcript levels in the SV under the different photoperiod treatments are similar to the changes in the Hyp. However, the expression levels of both *skissr* and *sGnRH3* are lower than those in the Hyp. This phenomenon was apparent both under different photoperiods and the different gonad development stages. Due to physiological activity of fish is too easily influenced by environment cues, we suggest that in the Atlantic salmon, the SV might be an organ which assists in regulating reproduction via photoperiodic signals to maintain normal physiological activity.

## Supporting information

S1 File Contains original figures and data. (RAR)
